# Dynamic analysis of recurrent phase patterns in spontaneous human EEG

**DOI:** 10.1186/1471-2202-14-S1-P157

**Published:** 2013-07-08

**Authors:** Won Sup Kim, Seung Kee Han

**Affiliations:** 1 Department of Physics, Chungbuk National University, Cheongju, Chungbuk 361-763, Korea

## 

Spontaneous human EEG during the resting state is modulating with long lasting high amplitude synchronization and very brief low amplitude de-synchronization states [1]. For the dynamic characterization of modulating alpha rhythm in the resting state EEG, we introduce a method of spontaneous-event related potential (SERP) analysis. Our method consists of three steps: At first, ensemble phase patterns of alpha rhythm at the moment of alpha de-synchronization state are classified using the K-mean clustering algorithm; Secondly, short time evolution of the phase pattern around each de-synchronization event is analyzed using the symbolic sequence dynamics; Finally, a global map of dynamic organization is constructed by integrating the temporal motifs representing the recurrent phase patterns around de-synchronization state.

Using the EEG data from seven subjects, very large number of de-synchronization event is collected from spike-like events in the time plot of inverse of alpha amplitude. The classification of the phase patterns of the de-synchronization state produces four different kinds of traveling waves, two propagating from posterior to anterior (PA_L _and PA_R_) and two in reverse directions (AP_L _and AP_R_) for C = 8 classification. The presence of two spiral waves, one rotating in clockwise (CS) and the other in count clockwise (CCS), are also observed in addition to two standing waves (ST_A _and ST_P_).

For the symbolic sequence analysis, we construct a triad symbol for each de-synchronization event. It is composed of a present pattern and its previous and next patterns, as a sequence of pre-present-post patterns. Then the occurrence rates of all possible triad symbols are compared with those of surrogate data where the sequence of all phase patterns is completely randomized. The triad symbols with very large normalized Z-score could be identified as dominant temporal motifs [2], which include the triad symbols, ST_P_-CCS-ST_A_, ST_P_-CS-ST_A_, CCS-ST_A_-AP_L_, and so on. We could also identify temporal anti-motifs as the triad symbols with very large negative Z-score. The anti-motifs include the triad symbols where strongly inhibited transitions like the transitions between CS and CCS, between ST_P _and two PA patterns, and between SP_A _and two AP patterns are included.

Integrating the information on the temporal motifs and anti-motifs, we could construct a global map of recurrent transition dynamic during the resting state. The global map contains the information on how the transitions among four traveling waves PA_L_, PA_R_, AP_L _and AP_R _occur. It is very interesting to notice the role played by the two spiral waves CS and CCS. As the motion of a spiral wave is recorded by tracing the phase singular point of a spiral wave, we observed that a traveling wave could switch its propagation as the spiral wave crosses the traveling wave in a transverse direction. This result indicates that the role of two spiral waves is to switch the propagation of traveling waves systematically.

In conclusion, using the SERP analysis of the spontaneous human EEG, we identified the recurrent phase patterns that involve the switching of traveling waves. Very interestingly, it is shown that the propagation of a traveling wave is systematically controlled by a spiral wave which drifts across the traveling wave. It is to be investigated on the function roles of the traveling waves and spiral waves, and also on the neural mechanisms of switching the propagation of the traveling waves [3] and the formation of spiral waves [4].
